# Anti‐envelope antibody responses in individuals at high risk of hepatitis C virus who resist infection

**DOI:** 10.1111/jvh.12568

**Published:** 2016-07-13

**Authors:** R. E. Swann, P. Mandalou, M. W. Robinson, M. M. Ow, S. K. H. Foung, J. McLauchlan, A. H. Patel, M. E. Cramp

**Affiliations:** ^1^ MRC – University of Glasgow Centre for Virus ResearchUniversity of GlasgowGlasgowUK; ^2^ Hepatology Research GroupPlymouth University Peninsula Schools of Medicine and DentistryPlymouthUK; ^3^ South West Liver UnitDerriford HospitalPlymouthUK; ^4^ School of Biochemistry and ImmunologyTrinity College DublinDublinIreland; ^5^ Department of PathologyStanford University School of MedicineStanfordCAUSA

**Keywords:** E1E2, exposed uninfected (EU), injection drug user (IDU), neutralization, neutralizing antibodies

## Abstract

Injection drug users uninfected by hepatitis C virus (HCV) despite likely repeated exposure through high‐risk behaviour are well documented. Factors preventing infection in these individuals are incompletely understood. Here, we looked for anti‐HCV‐envelope antibody responses in a cohort of repeatedly exposed but uninfected subjects. Forty‐two hepatitis C diagnostic antibody‐ and RNA‐negative injection drug users at high risk of exposure were studied and findings compared to healthy controls and cases with chronic HCV infection. Purified IgGs from sera were tested by ELISA for binding to genotype 1a and 3a envelope glycoproteins E1E2 with further testing for IgG and IgM reactivity against soluble E2. Virus‐neutralizing activity was assessed using an HCV pseudoparticle system. Uninfected subjects demonstrated significantly greater IgG and IgM reactivities to envelope glycoproteins than healthy controls with IgG from 6 individuals additionally showing significant neutralization. This study is the first to describe humoral immunological responses targeting the HCV envelope, important for viral neutralization, in exposed uninfected individuals. A subset of these cases also had evidence of viral neutralization via anti‐envelope antibodies. In addition to confirming viral exposure, the presence of specific anti‐envelope antibodies may be a factor that helps these individuals resist HCV infection.

## 1 Introduction

Hepatitis C virus (HCV) is a major cause of liver morbidity and mortality worldwide.^1^ Approximately 75% of infected individuals proceed to chronic infection.^2^ In developed countries, the major route of transmission remains the use of injection drugs and sharing of related paraphernalia by injection drug users (IDUs).^3^ Symptomatic acute infection is rare, and HCV has the potential to spread undetected within these populations. While effective antiviral medication is now available,^4^ treatment remains costly and ineffectively implemented with the prevalence of HCV in IDUs rising in England.^5^ It is clear that global eradication of HCV will not be possible unless robust preventative strategies are also developed.^6^ Studying individuals who resist infection can help inform prevention strategies and vaccine design.

In natural infection, successful clearance of HCV infection has been associated with the activation of elements of both the adaptive and the innate immune system (e.g. HCV‐specific B and T cell, NK cell, dendritic cell and interferon responses^7^, ^8^, ^9^, ^10^). There is growing evidence that a robust antibody response targeting HCV virion envelope glycoproteins (E1 and E2) responsible for virus entry into host cells can contribute significantly to viral clearance in acute infection.^11^, ^12^, ^13^, ^14^, ^15^, ^16^ Rapid onset of anti‐HCV envelope antibodies^17^ capable of neutralizing diverse strains of HCV is associated with acute clearance. Furthermore, broadly neutralizing antibodies may contribute to resolution of infection even once HCV has become established.^11^, ^18^ In the search for a prophylactic vaccine, it is especially relevant to study individuals who appear to have natural resistance to HCV infection. Individuals who regularly inject drugs and share injecting equipment are at very high risk of HCV exposure with seroprevalence rates of up to 90% reported in long‐term users. Resistance to HCV infection is increasingly well documented in IDUs who remain uninfected despite a long history of unsafe injection practices.^19^ Individuals at high risk of exposure with no evidence of past or current infection have been termed exposed but uninfected (EU).

EU cohorts are immunologically distinct from both healthy controls and those who spontaneously clear HCV infection (see Table 1).^20^, ^21^, ^22^ Up to 60% of EU individuals display HCV‐specific adaptive T‐cell responses. Furthermore, they display enhanced NK cell, IL6/IL8 and TNF‐α activity compared to HCV‐infected IDUs.^23^, ^24^ They are also genetically distinct from spontaneous resolvers and those with chronic HCV^9^ with the combination of KIR2DL3, HLA‐C1 over represented in both the EU and spontaneous resolver groups while the prevalence of the IL28B polymorphism is similar in EU to chronically infected cohorts.^22^, ^24^, ^25^, ^26^

**Table 1:** Immunological characteristics of exposed uninfected individuals

Immunological Component	Functional Outcome	Comparator Group	Reference
IL28B CC allele (rs12979860)	No difference compared to chronically infected individuals. Reduced frequency compared to spontaneous resolvers	Spontaneous resolvers and chronically infected individuals	9
KIR2DL3 genotype	Increased frequency of KIR2DL3:HLA‐C1 homozygosity	Chronically infected individuals	26
	No difference in frequency of KIR2DL3:HLA‐C1 homozygosity	Pre‐infection samples from IDUs with subsequent seroconversion	25
IL12B CC allele (rs3213113)	Increased frequency compared to healthy controls	Healthy controls	22
Serum cytokine levels	Elevated IL6, IL8 and TNF‐α	Healthy controls, spontaneous resolvers and chronically infected individuals	24
HCV‐specific T cells	Evidence of IFN‐γ production and T‐cell proliferation	Healthy controls and chronically infected individuals	21, 41
Natural Killer Cells	Enhanced IL‐2‐mediated cytotoxicity. Increased NKp30 expression	Pre‐infection samples from IDUs with subsequent seroconversion	23

While this EU cohort is defined by the lack of antibody response to HCV core and nonstructural proteins in a diagnostic assay, the role that anti‐envelope neutralizing antibodies might play in their protection from infection has not yet been explored. Given the upregulation of neutralizing antibodies in those who acutely clear HCV infection on multiple exposures, it is possible that such antibodies may have a significant protective effect in the IDU EU population.^12^ Therefore, we aimed to determine the presence of functional anti‐HCV envelope antibody responses in a cohort of IDUs who remain uninfected despite their high risk of repeated exposure to HCV.

## 2 Materials and methods

### 2.1 The EU and control cohorts

A cohort of current IDU not known to have HCV infection or other blood borne viruses was recruited between 2003 and 2014 from a variety of locations in Plymouth, UK, as previously described.^21^, ^22^ All individuals completed a confidential structured questionnaire to collect demographic data and a detailed injecting history. This included age at first injection, duration of injecting behaviour, frequency of injecting episodes, current injecting behaviour, frequency of sharing intravenous paraphernalia (needles, syringes, filters, spoons and water), frequency of sharing with a contact known to have HCV infection and risk of non‐IDU HCV exposure. For this study, we included only those judged to be at substantial risk of HCV exposure based on a >1 year history of injecting drug use and regular sharing of needles and related paraphernalia. All individuals had serum and whole blood samples drawn for laboratory analysis and clinical details entered in respective databases. Where possible, follow‐up clinical information and clinical samples from recruited individuals were obtained.

The exposed group was screened for the evidence of current or previous HCV infection using diagnostic tests for antibodies to core and nonstructural proteins (third‐generation ELISA by Abbot IMx) and HCV RNA by qualitative PCR (Amplicor, Roche Diagnostics). Those individuals negative on both tests were termed exposed uninfected (EU) and were included in the study. Two individuals who were found to have developed HCV infection on subsequent testing were excluded. In addition, we separately recruited positive and negative control cohorts of individuals with chronic HCV infection (CHCV) and non‐IDU healthy controls (HC) with no liver disease or history of HCV infection, respectively. The full details of these cohorts have been described elsewhere.^27^

Study protocols conformed to the ethical guidelines of the 1975 Declaration of Helsinki as reflected in *a priori* approval by regional ethical committees. Informed written consent was obtained from each subject prior to entry in the study.

### 2.2 Statistical analysis

Statistical analysis was conducted using GraphPad Prism 6 Software (GraphPad Software, California, USA) and SPSS v. 19.09 (IBM, New York, USA).

### 2.3 Generation of HCV pseudoparticle (HCVpp) and E1E2 Lysate

As genotypes 1 (gt 1) and 3 (gt 3) account for the vast majority of HCV infections in the UK, envelope glycoprotein sequences from a standard HCV gt 1a and a UK derived gt 3 strain were used. HCV pseudoparticles bearing envelope proteins from gt 1a (strain H77c, accession number AF011751.1) and gt 3a (sequence UKN3a1.28 F4/2‐35; closely related to accession number AY734984.1) were generated in HEK‐293T cells as described previously.^28^ Further details are available in Supplementary Methods. Following harvesting of pseudoparticles, cells were lysed in 1 mL of lysis buffer and centrifuged and the supernatant used for E1E2 ELISA assays as described below.

### 2.4 GNA capture E1E2 ELISA

IgG was purified from EU, CHCV and HC samples using Protein G IgG‐specific spin columns (Thermo Scientific, UK; see Supplementary Methods) including 3 wash steps prior to elution ensuring removal of nonspecific serum factors that may interfere with the assay. Purified IgG was tested in an ELISA assay to detect antibodies to the E1 and E2 glycoproteins contained in the HEK‐293T lysate supernatant. These ELISAs were performed as described previously with further detail in Supplementary Methods.^29^ Absorbance readings were normalized to a multiple of the mean of the HC readings. Significant binding by ELISA was determined as absorbance values ≥2 times HC mean. Reactivity to both gt 1a and gt 3a E1E2 was tested.

### 2.5 Soluble gt 1a E2 binding ELISAs: IgG and IgM

As IgM is difficult to purify, diluted serum was used to detect binding of IgG and IgM to purified HCV soluble gt 1a E2 (sE2). EU and control serum samples were diluted 1:50 in PBSTM and tested for binding to sE2 by ELISA. Absorbance readings were normalized to healthy control mean and analysed as above with further detail in the Supplementary Methods.

### 2.6 Pseudoparticle neutralization assays

In the subset of EU individuals displaying significantly elevated E1E2 binding compared to HC on ELISA, pseudoparticle (pp) neutralization assays were conducted as previously described.^30^ Briefly, purified subject IgG was added to 40 μL of HCVpp‐containing medium prepared as above. Purified IgG was added at a concentration of 400 μg mL^−1^ for screening of the EU cohort, for some individuals where serum was scarce, this was reduced to 200 μg mL^−1^. The mouse monoclonal anti‐E2 antibody AP33^31^ and HC IgG were included as positive and negative controls, respectively. After incubation for 1 hour, the IgG‐HCVpp mixture was added to a 96‐well plate preseeded with Huh‐7 cells. Following incubation for 3 hour, the inoculum was replaced with fresh medium, and after a further 72‐hour incubation, luciferase activity in infected cells was detected as a marker of HCVpp infectivity using the Bright‐Glo Luciferase kit (Promega, UK). Virus neutralization was defined as 50% reduction in pseudoparticle infectivity as measured in relative light units (RLU) using a Chameleon II plate reader. Ability to neutralize both gt 1a and gt 3 was tested. For those samples with apparent neutralizing activity, further neutralization assays were conducted using serial dilutions of subject IgG.

### 2.7 E2 competition ELISA

For EUs with neutralizing activity and sufficient IgG available, competition ELISAs were performed using a panel of well‐characterized monoclonal antibodies targeting known CD81‐binding epitopes (Table S1). CHCV samples with known neutralizing activity were used as positive controls.

Purified IgG was incubated on E2‐coated Immulon II plates at a concentration of 200 μg mL^−1^ in PBSTM. Subsequently, biotinylated antibodies to known conformational epitopes were added to these wells in addition to control wells with no EU IgG. Streptavidin‐HRP was added and binding measured as previously. The reduction in relative binding of each biotinylated antibody (calculated as percentage reduction in absorbance) on addition of EU IgG compared to control was determined. PBSTM and neutralizing IgG from the CHCV cohort were used as negative and positive controls, respectively.

## 3 Results

### 3.1 Clinical cohorts

Forty‐two EU subjects, 8 gt 1 chronically infected patients (CHCV) and 8 healthy controls (HC) were studied. By definition, all EU cases consistently tested negative for HCV RNA and anti‐HCV antibodies using standard diagnostic tests as described. In 5 EU individuals, serial samples were tested. Demographics of the cohorts are detailed in Table 2.

**Table 2:** Demographics of clinical cohorts tested

	Exposed uninfected (EU)n=42	Chronic HCV gt 1 (CHCV)n=8^a^	Healthy controls (HC)n=8
Mean age at sampling	34±8.3	52±6.3	47±18.2
Ethnicity (% Caucasian)	100	100	100
Sex (% Male)	89	63	50
Current IDU (%)	100	–	0
Mean age at commencing IDU (years±SD)	21.8±5.2	ND	N/A
Mean lifetime injecting episodes (range)	4128 (52–21 900)	ND	N/A
Duration of IDU (years±SD)	7.6±4.3	ND	N/A
Sharing needles/syringes (%)	72	ND	N/A
Sharing any injection equipment (%)	100	ND	N/A
Sharing with IDU known to suffer from HCV infection (%)	28.5	ND	N/A

ND, not determined; N/A, not applicable.

a The majority of CHCV patients listed their likely source of infection as IDU; however, detailed data on historical and present injected behaviour were not collected. Within the CHCV group, the estimated median duration of infection was 31 years, with all individuals having been infected for a minimum of 6 months prior to study recruitment.

### 3.2 Reactivity of IgG to HCV envelope

**Figure 1:**
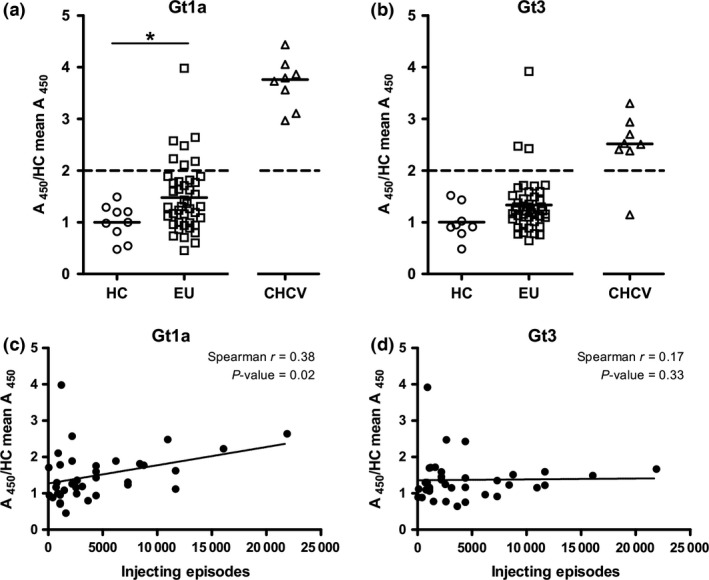
EU individuals show elevated IgG reactivity to HCV envelope proteins which correlates with lifetime risk of exposure. (a+b) Purified IgGs were tested for their ability to bind HEK‐293T‐expressed E1E2 in a (**a**) GNA capture gt 1a and (**b**) GNA capture gt 3a ELISAs. For those individuals where serum from multiple time points was available, each time point was tested individually, but only one value for each individual (i.e. the first sample taken) is plotted. Statistical differences between the groups were calculated using the Wilcoxon rank sum test (*P<.05). (c+d) Correlation between IgG reactivity to either gt 1a (**c**) or gt 3a (**d**) and total number of lifetime injecting episodes (duration of reported injection drug use multiplied by the frequency of reported injection drug use) was plotted for 37 EU (injection data not available for the remaining 5)

The median absorbance readings for the HCV gt 1a E1E2 capture ELISA were significantly higher in the EU cohort than in the HC group (*P*=.038) (Fig. 1a). While there was a trend towards overall higher absorbance readings against gt 3a E1E2 in the EU group than in controls, this did not reach significance (*P*=.067, Fig. 1b). However, three samples showed levels of binding to gt 3a lysate comparable to chronically infected controls.

### 3.3 HCV exposure risk and E1E2 reactivity

EU IgG absorbance levels to gt 1a E1E2 and gt 3a E1E2 were analysed for association with reported injection behaviour with a significant correlation between IgG reactivity against gt 1a E1E2 and greater lifetime injecting episodes (Spearman *R*=.38, *P*=.02, Fig. 1c), although this association was not seen with gt 3a reactivity (Fig. 1d).

### 3.4 IgG and IgM sE2 ELISA

**Figure 2:**
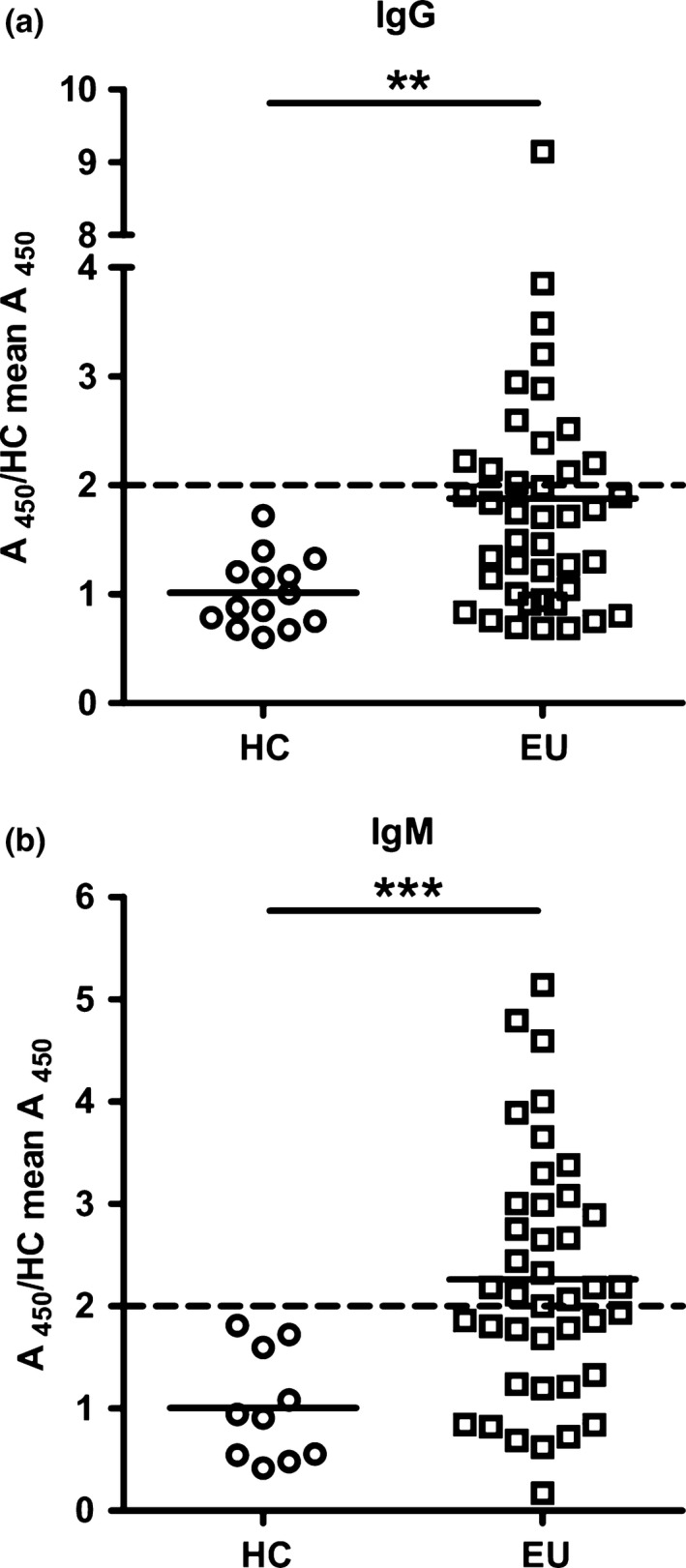
IgG and IgM responses to soluble gt 1a E2 are evident in EU individuals. IgG (**a**) and IgM (**b**) absorbance to purified gt1a sE2 protein was determined for EU and HC individuals using a modified ELISA protocol described in the Supplementary Methods. Statistical differences between the groups were calculated using the Wilcoxon rank sum test. **P<.01, *** *P*<.001

To eliminate reactivity against HEK antigens and medium components, diluted serum samples from all individuals were tested for IgG reactivity to gt 1a sE2. For two of the subjects, serum from 2 separate time points was tested. There was significantly higher absorbance of IgG from EU to sE2 than HC (*P*<.01, Fig. 2a). Serum was also tested for IgM binding to sE2. The EU cohort showed significantly higher quantities of IgM binding to sE2 than the HC group (*P*=.001 Wilcoxon rank sum, Fig. 2b) with several individuals displaying levels comparable to the CHCV group (data not shown).

### 3.5 Positivity across multiple assays

We set a cut‐off of absorbance ≥2 times the HC mean value as indicating a significantly positive result. Overall, 20 of 42 (47%) EU showed evidence of IgG reactivity to HCV envelope E1E2 proteins on either the GNA capture ELISA or IgG gt 1a purified soluble E2 (sE2) binding ELISA using this cut‐off (Table S2). Four of the 7 EUs with ≥2 times the HC mean binding to gt 1a E1E2 lysate also had significant binding to sE2 displaying absorbance readings ≥2 times the HC mean (Fig. 2a, Table S2) with 2 more showing above average binding to sE2 but below this cut‐off value. Seven further individuals with significant responses against sE2 showed elevated reactivity against gt 1a lysate, but the absorbance attained did not reach the cut‐off value. Only four samples showed significant binding to sE2 alone. It should be noted that for the vast majority of assays the value of 2 times the HC mean was higher (therefore a more stringent cut‐off) than HC mean + 2 times standard deviations.

### 3.6 Ability to neutralize HCVpp

**Figure 3:**
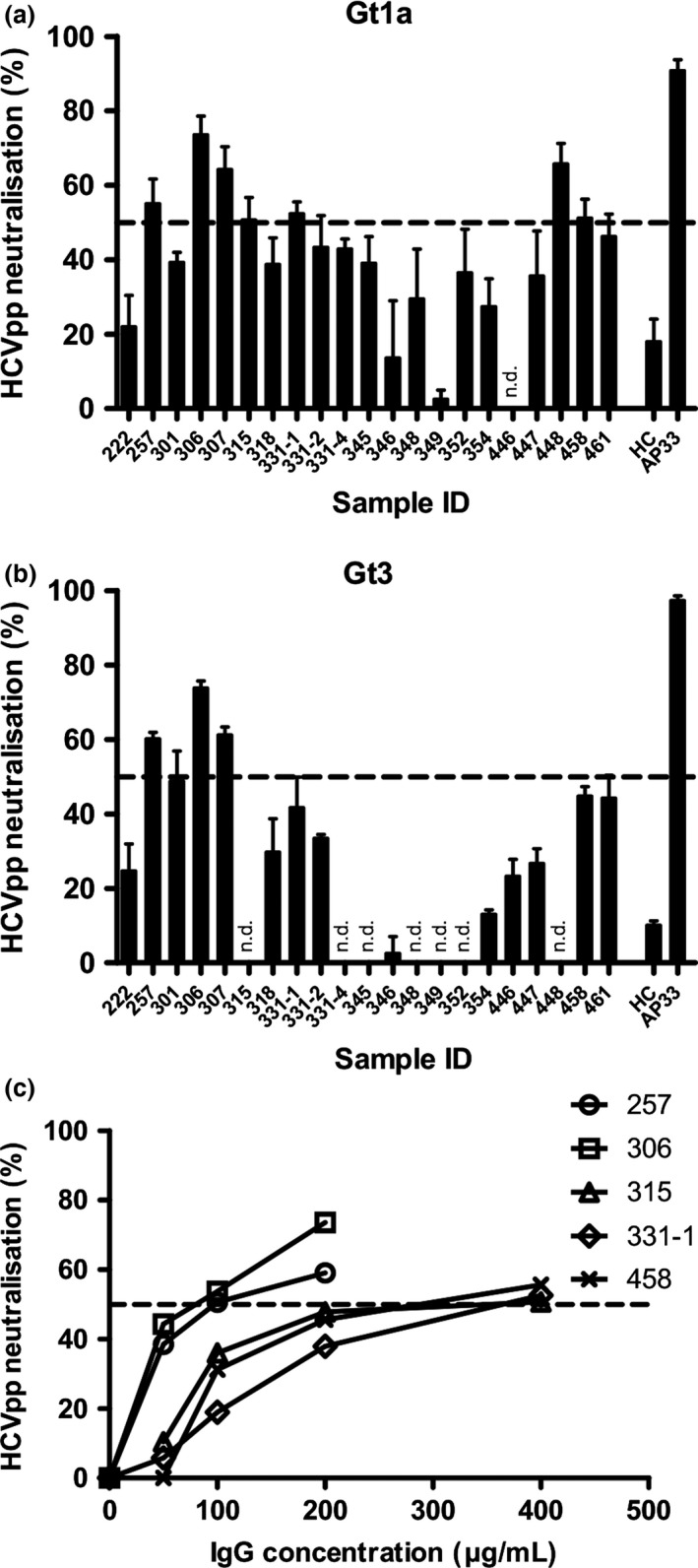
Neutralization of HCVpp by purified IgGs from EU. Virus pseudoparticle neutralization assays were performed using HCVpp bearing E1E2 derived from (**a** and **c**) gt 1a strain H77 or (**b**) gt 3 HCV. (**a** and **b**) Percentage reduction in HCVpp entry after incubation with 400 μg mL ^−1^ test IgG is plotted as evidenced by luciferase reading at 72 hours compared to control. (**c**) Percentage reduction in HCVpp entry after incubation with reducing concentrations of IgG as shown. The 50% cut‐off is shown as a dashed lined. All results represent the average of at least 3 separate technical replicates, with error bars representing the SEM

Of those EU subject sera with significant binding to E1E2 by ELISA, 6 (257, 306, 307, 315, 331‐1 and 458) showed ability to neutralize gt 1a HCVpp by 50% or more at a purified IgG concentration of 400 μg mL^−1^ (Fig. 3a). For subjects 257, 306 and 307, this level of neutralization was achieved at a concentration of 200 μg mL^−1^, while IC_50_ value for the remaining samples was between 200 μg mL^−1^ and 400 μg mL^−1^ (Fig. 3c).

Of those able to neutralize gt 1a HCVpp, 3 EU individuals (257, 306 and 307) were also able to reduce infectivity of gt 3a HCVpp by 50% or greater with one further gt 1a neutralizing subject reducing gt 3a infectivity by 40% (EU 458) (Fig. 3b). Two further individuals (461, 301) showed evidence of a weaker neutralization effect against both gt 1a and gt 3a HCVpp, consistently reducing infectivity by 40%.

### 3.7 Competition with antibodies to known epitopes

**Figure 4:**
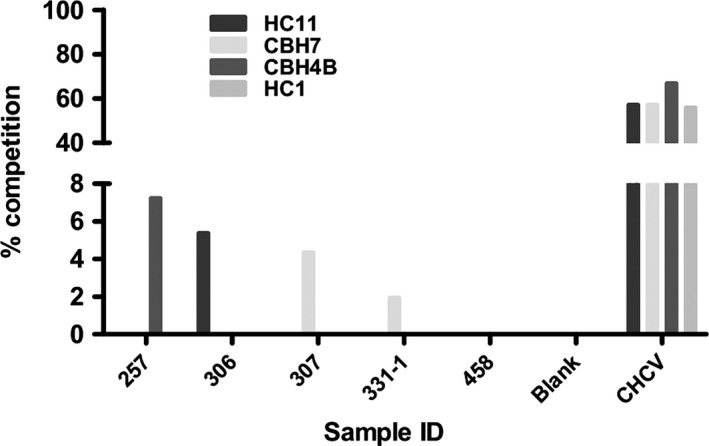
EU IgG fails to compete with conformational antibodies to known epitopes on gt 1a E2. IgGs from individuals with neutralizing ability in the HCV pp system were selected for testing by competition ELISA to determine competitive binding with monoclonal antibodies to known conformational epitopes on E2 (Table S1). Samples from chronically infected individuals (CHCV) with known neutralizing activity were also included as positive controls. Percentage reduction in absorbance of the monoclonal antibodies was calculated and plotted. Significant competition would be expected at a level of 50% inhibition

IgG from EU individuals with evidence of neutralizing activity was tested in a competition assay with a small panel of well‐characterized conformation‐sensitive anti‐E2 antibodies. Three of these (HC1, HC11 and CBH7) recognize amino acid residues/regions that are critical for the interaction of E2 to CD81, the host entry factor essential for virus entry into target cells^13^, ^14^, ^15^, ^16^ (Table S1). Although chronically infected samples with neutralizing activity tended to compete with antibodies to these regions, we did not see any evidence of significant competition in the EU samples (Fig. 4). While there was evidence of binding to pure E2 in these samples at >2× control samples (Fig. 2a), it was considerably weaker than in individuals with chronic infection which bound to viral glycoproteins at >20 times the strength of HC samples (data not shown).

### 3.8 Duration of antibody detection over time

For 5 EU individuals, serial samples from time points separated by a period of at least 1 year were studied. Their E1E2 reactivity values relative to HC samples were consistent over the time periods studied, either remaining at HC levels or being consistently elevated (Fig. S1). In the one individual with significant E1E2 and neutralizing responses studied serially, these responses remained detectable over time but with a diminution in strength at later time points.

## 4 Discussion

Long‐term injection drug users who recurrently, persistently and frequently share injection equipment but who remain HCV antibody negative with undetectable HCV RNA represent an optimal cohort for advancing knowledge and understanding of mechanisms of natural protection from HCV infection. These individuals appear resistant to infection. Unlike HIV where resistance may be conferred by mutations in host entry proteins, no such variants have been identified in HCV exposed uninfected cases.^32^, ^33^, ^34^ Therefore, it is likely that these subjects resist infection through immune‐mediated mechanisms.

This study is the first to report evidence of humoral immunological responses targeting the HCV envelope, potentially a key area for viral neutralization, in exposed uninfected individuals. While previous research has identified HCV‐specific T‐cell responses in EU individuals, it remains unclear if those responses are able to protect from infection or, perhaps more likely, are merely a marker of viral exposure. This is supported by the fact that HCV‐specific T‐cell responses are generally weak and wane rapidly on the cessation of injection drug use.^21^, ^35^ The presence of HCV envelope antibody responses provides further confirmation that this EU group has indeed been exposed to HCV infection, but whether the humoral response is robust enough to have a role in providing protection remains unanswered.

Anti‐HCV humoral responses are not detected by diagnostic antibody assays in this cohort who by definition remain seronegative by conventional testing. However, it must be remembered that current commercial diagnostic assays do not detect anti‐envelope antibodies. We have identified anti‐E1E2 IgG responses at levels significantly higher than controls in many EU cases with anti‐E1E2 IgG binding at levels >2 times healthy controls in at least one assay observed in 20 of the 42 EU cases. While such a cut‐off is arbitrary and risks false‐positive (and false‐negative) results, all those with neutralizing activity show binding around this level supporting use of this cut‐off.

Furthermore, IgGs from 6 of our cohort were able to reduce HCVpp entry into hepatocytes *in vitro* by 50% at IC50s ranging from 75 to 400 mg mL^−1^ (equating to a serum dilution of 1:100–1:25) with other EU individuals demonstrating a weaker neutralizing response. These are comparable to the strength of neutralizing responses observed in spontaneous resolving patients during viral clearance.^36^ It is precisely this group of individuals that should be targeted in efforts to identify protective E1E2 epitopes for vaccine development.

Primate studies using HCV gt 1a envelope proteins as a vaccine immunogen have shown that neutralizing antibodies were raised in the majority of animals immunized on 2 occasions. These were able to prevent *de novo* infection and had some activity against strains of virus from different genotypes.^37^ In our cohort, in addition to serving as a marker of viral exposure, the presence of anti‐envelope antibodies suggests one possible mechanism by which these subjects may have resisted infection. This is further supported by the *in vitro* evidence for viral neutralization with IgGs from a subset of individuals significantly reducing cellular entry by HCVpp.

In those who resolve acute infection, detectable neutralizing antibody responses are seen to reduce over a period of months to years following viral clearance although they may be restored in subsequent episodes of infection to aid more rapid viral control.^12^ The presence of potentially protective humoral responses in our cohort is seen most strikingly in cases with the greatest likelihood and frequency of HCV exposure. One individual in our cohort (331) showed the persistence of antibody responses over a period of a year. It remains to be determined if anti‐envelope antibody responses need ongoing priming by exposure to HCV to be sustained or whether the responses demonstrated could provide ongoing protection on cessation of IDU.

In order to establish whether there was evidence of recent exposure to HCV envelope proteins, we looked for the evidence of IgM response to E2. While anti‐HCV IgM persists in chronic infection, it wanes with viral clearance, with levels declining 8 weeks following exposure.^38^, ^39^ The elevated levels of anti‐E2 IgM observed in our EU subjects likely reflect an immune response to ongoing intermittent exposure to envelope proteins as might be expected through high‐risk injecting practices. The strength of these responses was equivalent to those seen in chronically infected individuals, and the precise role of IgM in resisting infection in the EU cohort warrants further study.

While we have been able to show a neutralizing effect of EU IgG, the regions of the viral glycoprotein targeted by these antibodies have yet to be defined. The absence of competition with antibodies targeting known E2 epitopes raises the possibility that novel epitopes are involved. However, as avidity of binding increases with the duration of infection,^40^ it is also conceivable that the EU antibody responses are directed at CD81 binding regions in E2 but do not bind with sufficient avidity to avoid being ‘competed off’ by the conformational antibodies derived from chronically infected individuals. It is also possible that these individuals, in common with most acutely infected cases, develop antibodies which predominantly target the Highly Variable Region‐1 region of the E2 protein or regions of E1 essential for entry. An alternative explanation may be that due to high levels of diversity in E2 amino acid sequences, these individuals may raise antibodies to a local E2 sequence which only provides weak cross‐reactivity with the H77 sequence at CD81 binding epitopes, and future work should aim to explore the breadth of E1E2 reactivity within these individuals.

In conclusion, this is the first report of HCV envelope‐specific humoral immune responses in a cohort of exposed uninfected injection drug users who remain HCV PCR and EIA negative despite repeated risk of exposure. In addition to adaptive humoral immune responses to envelope proteins, some individuals produce neutralizing anti‐envelope antibodies which may contribute to host immunity. These neutralizing antibodies do not appear to compete with commonly targeted CD81 binding sites and may therefore recognize novel epitopes. Our data complement previous reports of HCV‐specific T‐cell responses^9^, ^26^ and upregulated innate immune responses in exposed uninfected cases.^25^ Together, these studies provide robust evidence that such individuals have been exposed to HCV, but are resistant to developing established infection. Whether any one of these responses is central to resisting infection, or whether a combination of upregulated innate immunity together with adaptive T‐ and B‐cell responses is needed is not yet known. Further study of the antibody responses present in EU individuals will enhance our understanding of the role of antibodies in HCV infection and inform future preventative vaccine design.

## Acknowledgements and disclosures

We thank all those who kindly provided blood samples for study, and the community drug services who helped us identify and recruit exposed uninfected cases. We thank Francois‐Loıc Cosset and Jonathan Ball for the provision of reagents; and Ania Owsianka, Sarah Cole and Vanessa Cowton for their valuable advice and help with experiments. We also thank Medical Research Council, UK (Grant Reference MC_UU_12014/2 and Clinical Research Fellowship Grant No: G0801822) and Plymouth Hospitals NHS Trust, South West Liver Unit for funding this study.

## Statement of interests

The authors report no conflict of interests relevant to this manuscript. No external assistance was sought in the writing of this manuscript.

**Abbreviations**
CHCV: chronically HCV‐infected Cohort
E1E2: envelope glycoproteins 1 and 2
E2: envelope glycoprotein E2
EIA: enzyme Immunoassay
EU: exposed uninfected
Gt: genotype
HC: healthy control cohort
HCV: hepatitis C Virus
HCVpp: hepatitis C Virus pseudoparticles
IDUs: injection drug users
PBSTM: PBS containing 0.05% Tween‐20 and 2% Skimmed Milk Powder
sE2: purified soluble envelope protein E2

## References

[1] WHO . WHO fact sheet: HCV vaccines number 164. 2012 http://www.who.int/mediacentre/factsheets/fs164/en/. Accessed 06 July 2016.

[2] Bowen DG , Walker CM . Adaptive immune responses in acute and chronic hepatitis C virus infection. Nature 2005;436:946–952. 16107834 10.1038/nature04079

[3] Law MG , Dore GJ , Bath N , et al. Modelling hepatitis C virus incidence, prevalence and long‐term sequelae in Australia, 2001. Int J Epidemiol. 2003;32:717–724. 14559738 10.1093/ije/dyg101

[4] Kim DY , Ahn SH , Han KH . Emerging therapies for hepatitis C. Gut Liv. 2014;8:471–9. 10.5009/gnl14083 PMC4164256 25228970

[5] Sweeting MJ , Hope VD , Hickman M , et al. Hepatitis C infection among injecting drug users in England and Wales (1992–2006): there and back again? Am J Epidemiol. 2009;170:352–360. 19546152 10.1093/aje/kwp141 PMC2714950

[6] Hagan LM , Schinazi RF . Best strategies for global HCV eradication. Liver Int. 2013;33(Suppl 1):68–79. 23286849 10.1111/liv.12063 PMC4110680

[7] Thimme R , Binder M , Bartenschlager R . Failure of innate and adaptive immune responses in controlling hepatitis C virus infection. FEMS Microbiol Rev 2012;36:663–683. 22142141 10.1111/j.1574-6976.2011.00319.x

[8] Thimme R , Neumann‐Haefelin C , Boettler T , Blum HE . Adaptive immune responses to hepatitis C virus: from viral immunobiology to a vaccine. Biol Chem. 2008;389:457–467. 18953713 10.1515/bc.2008.061

[9] Knapp S , Warshow U , Ho KM , et al. A polymorphism in IL28B distinguishes exposed, uninfected individuals from spontaneous resolvers of HCV infection. Gastroenterology 2011;141:320–325, 5 e1‐2. 21600205 10.1053/j.gastro.2011.04.005 PMC3194089

[10] Thomas DL , Thio CL , Martin MP , et al. Genetic variation in IL28B and spontaneous clearance of hepatitis C virus. Nature 2009;461:798–801. 19759533 10.1038/nature08463 PMC3172006

[11] Raghuraman S , Park H , Osburn WO , Winkelstein E , Edlin BR , Rehermann B . Spontaneous clearance of chronic hepatitis C virus infection is associated with appearance of neutralizing antibodies and reversal of T‐cell exhaustion. J Infect Dis. 2012;205:763–771. 22293431 10.1093/infdis/jir835 PMC3274373

[12] Osburn WO , Fisher BE , Dowd KA , et al. Spontaneous control of primary hepatitis C virus infection and immunity against persistent reinfection. Gastroenterology 2010;138:315–324. 19782080 10.1053/j.gastro.2009.09.017 PMC2889495

[13] Bartosch B , Cosset FL . Cell entry of hepatitis C virus. Virology 2006;348:1–12. 16455127 10.1016/j.virol.2005.12.027

[14] Hamilton JP , Thuluvath PJ . Claudin‐1 and its potential role in HCV entry: another piece of the puzzle. J Clin Gastroenterol. 2008;42:3–4. 18097281 10.1097/MCG.0b013e31814a4e7d

[15] Meredith LW , Wilson GK , Fletcher NF , McKeating JA . Hepatitis C virus entry: beyond receptors. Rev Med Virol. 2012;22:182–193. 22392805 10.1002/rmv.723

[16] Ploss A , Evans MJ . Hepatitis C virus host cell entry. Curr Opin Virol. 2012;2:14–19. 22440961 10.1016/j.coviro.2011.12.007 PMC3311996

[17] Pestka JM , Zeisel MB , Blaser E , et al. Rapid induction of virus‐neutralizing antibodies and viral clearance in a single‐source outbreak of hepatitis C. Proc Natl Acad Sci USA 2007;104:6025–6030. 17392433 10.1073/pnas.0607026104 PMC1851610

[18] de Jong YP , Dorner M , Mommersteeg MC , et al. Broadly neutralizing antibodies abrogate established hepatitis C virus infection. Sci Transl Med 2014;6:254ra129. 10.1126/scitranslmed.3009512 PMC4312107 25232181

[19] Mina MM , Luciani F , Cameron B , et al. Resistance to hepatitis C virus: potential genetic and immunological determinants. Lancet Infect Dis. 2015;15:451–460. 25703062 10.1016/S1473-3099(14)70965-X

[20] Cramp ME , Carucci P , Rossol S , et al. Hepatitis C virus (HCV) specific immune responses in anti‐HCV positive patients without hepatitis C viraemia. Gut. 1999;44:424–429. 10026332 10.1136/gut.44.3.424 PMC1727419

[21] Thurairajah PH , Hegazy D , Chokshi S , et al. Hepatitis C virus (HCV)–specific T cell responses in injection drug users with apparent resistance to HCV infection. J Infect Dis. 2008;198:1749–1755. 18959498 10.1086/593337

[22] Hegazy D , Thurairajah P , Metzner M , et al. Interleukin 12B gene polymorphism and apparent resistance to hepatitis C virus infection. Clin Exp Immunol. 2008;152:538–541. 18422730 10.1111/j.1365-2249.2008.03655.x PMC2453209

[23] Golden‐Mason L , Cox AL , Randall JA , Cheng L , Rosen HR . Increased natural killer cell cytotoxicity and NKp30 expression protects against hepatitis C virus infection in high‐risk individuals and inhibits replication in vitro. Hepatology 2010;52:1581–1589. 20812318 10.1002/hep.23896 PMC2967665

[24] Warshow UM , Riva A , Hegazy D , et al. Cytokine profiles in high risk injection drug users suggests innate as opposed to adaptive immunity in apparent resistance to hepatitis C virus infection. J Viral Hepat. 2012;19:501–508. 22676363 10.1111/j.1365-2893.2011.01574.x

[25] Sugden PB , Cameron B , Luciani F , Lloyd AR , Investigators H . Exploration of genetically determined resistance against hepatitis C infection in high‐risk injecting drug users. J Viral Hepat. 2014;21:e65–73. 24612442 10.1111/jvh.12232

[26] Knapp S , Warshow U , Hegazy D , et al. Consistent beneficial effects of killer cell immunoglobulin‐like receptor 2DL3 and group 1 human leukocyte antigen‐C following exposure to hepatitis C virus. Hepatology 2010;51:1168–1175. 20077564 10.1002/hep.23477 PMC4202114

[27] Robinson MW , Swann R , Sigruener A , et al. Elevated interferon‐stimulated gene transcription in peripheral blood mononuclear cells occurs in patients infected with genotype 1 but not genotype 3 hepatitis C virus. J Viral Hepat. 2014;22:384–390. 25200131 10.1111/jvh.12310 PMC4409080

[28] Bartosch B , Bukh J , Meunier JC , et al. In vitro assay for neutralizing antibody to hepatitis C virus: evidence for broadly conserved neutralization epitopes. Proc Natl Acad Sci U S A. 2003;100:14199–14204. 14617769 10.1073/pnas.2335981100 PMC283569

[29] Patel AH , Wood J , Penin F , Dubuisson J , McKeating JA . Construction and characterization of chimeric hepatitis C virus E2 glycoproteins: analysis of regions critical for glycoprotein aggregation and CD81 binding. J Gen Virol. 2000;81:2873–2883. 11086118 10.1099/0022-1317-81-12-2873

[30] Keck ZY , Li TK , Xia J , et al. Analysis of a highly flexible conformational immunogenic domain a in hepatitis C virus E2. J Virol. 2005;79:13199–13208. 16227243 10.1128/JVI.79.21.13199-13208.2005 PMC1262592

[31] Owsianka A , Clayton RF , Loomis‐Price LD , McKeating JA , Patel AH . Functional analysis of hepatitis C virus E2 glycoproteins and virus‐like particles reveals structural dissimilarities between different forms of E2. J Gen Virol. 2001;82:1877–1883. 11457993 10.1099/0022-1317-82-8-1877

[32] Michael NL , Louie LG , Rohrbaugh AL , et al. The role of CCR5 and CCR2 polymorphisms in HIV‐1 transmission and disease progression. Nat Med. 1997;3:1160–1162. 9334732 10.1038/nm1097-1160

[33] Deest M , Westhaus S , Steinmann E , Manns MP , von Hahn T , Ciesek S . Impact of single nucleotide polymorphisms in the essential HCV entry factor CD81 on HCV infectivity and neutralization. Antiviral Res. 2014;101:37–44. 24211330 10.1016/j.antiviral.2013.10.018

[34] Houldsworth A , Metzner MM , Demaine A , Hodgkinson A , Kaminski E , Cramp M . CD81 sequence and susceptibility to hepatitis C infection. J Med Virol. 2014;86:162–168. 24122777 10.1002/jmv.23726

[35] Cameron B , Galbraith S , Li H , Lloyd A , Investigators H . Correlates and characteristics of hepatitis C virus‐specific T‐cell immunity in exposed uninfected high‐risk prison inmates. J Viral Hepat 2013;20:e96–106. 23490396 10.1111/jvh.12016

[36] Esteban‐Riesco L , Depaulis F , Moreau A , et al. Rapid and sustained autologous neutralizing response leading to early spontaneous recovery after HCV infection. Virology. 2013;444:90–99. 23890816 10.1016/j.virol.2013.05.037

[37] Meunier JC , Gottwein JM , Houghton M , et al. Vaccine‐induced cross‐genotype reactive neutralizing antibodies against hepatitis C virus. J Infect Dis. 2011;204:1186–1190. 21917891 10.1093/infdis/jir511 PMC3203383

[38] Clemens JM , Taskar S , Chau K , et al. IgM antibody response in acute hepatitis C viral infection. Blood. 1992;79:169–172. 1309424

[39] Brillanti S , Masci C , Miglioli M , Barbara L . Serum IgM antibodies to hepatitis C virus in acute and chronic hepatitis C. Arch Virol Supplementum. 1993;8:213–218. 10.1007/978-3-7091-9312-9_21 7505144

[40] Gaudy‐Graffin C , Lesage G , Kousignian I , et al. Use of an anti‐hepatitis C virus (HCV) IgG avidity assay to identify recent HCV infection. J Clin Microbiol. 2010;48:3281–3287. 20610669 10.1128/JCM.00303-10 PMC2937703

[41] Mizukoshi E , Eisenbach C , Edlin BR , et al. Hepatitis C virus (HCV)‐specific immune responses of long‐term injection drug users frequently exposed to HCV. J Infect Dis. 2008;198:203–212. 18505381 10.1086/589510 PMC2699613

