# Supplementary material for: Anti‐envelope antibody responses in individuals at high risk of hepatitis C virus who resist infection
Source: J Viral Hepat. 2016 Jul 13;23(11):873–80. doi: 10.1111/jvh.12568 (PMC5244678; doi:10.1111/jvh.12568)
Supplement: Supplementary file 1 [file JVH-23-873-s001.docx]

**Anti-envelope antibody responses in individuals at high risk of hepatitis C virus who resist infection.**

Rachael E. Swann,^1,†^ Paraskevi Mandalou,^2,†^ Mark W. Robinson,^1,3^ Maggie M Ow,^2^ Steven K. H. Foung,^4^ John McLauchlan,^1^ Arvind H. Patel,^1,*^ and Matthew E. Cramp^2,*^

^†^Both authors contributed equally to the work and are joint first authors

^1^MRC - University of Glasgow Centre for Virus Research, University of Glasgow, G61 1QH, UK.

^2^Hepatology Research Group, Plymouth University Peninsula Schools of Medicine and Dentistry and South West Liver Unit, Derriford Hospital, Plymouth, PL6 8DH UK.

^3^ School of Biochemistry and Immunology, Trinity College Dublin, Dublin, Ireland.

^4^Department of Pathology, Stanford University School of Medicine, Stanford, California, USA

^*^ **Corresponding Authors:**

Prof. Matthew E. Cramp, Hepatology Research Group, South West Liver Unit, Derriford Hospital, Plymouth, UK, PL6 8DH. **email** – [matthew.cramp@nhs.net](mailto:matthew.cramp@nhs.net)

Dr Arvind H. Patel, MRC - University of Glasgow Centre for Virus Research, University of Glasgow, Glasgow, G61 1QH, UK. Tel: +44 141 330 4026; Fax: +44 141 330 2271.

**email** – [Arvind.patel@glasgow.ac.uk](mailto:Arvind.patel@glasgow.ac.uk)

**Supplementary Material:**

**Table of Contents:**

**Supplementary Methods**

***Serum preparation and IgG purification***

***Cell lines***

***Generation of HCV pseudoparticles***

***E1E2 GNA Capture ELISA***

***E1E2 GNA Capture ELISA***

**Supplementary Fig. 1.** **Anti-E1E2 responses over time.**

**Supplementary** **Table 1: Binding sites of conformational antibodies used in competition ELISA**

**Supplementary Table 2: IgG ELISA and Neutralization assay results for individual EU subjects**

**Supplementary Methods:**

***Serum preparation and IgG purification***

Serum was incubated with Triton X-100 (Sigma, UK) at a final concentration of 0.05% to deactivate any virus particles present. Disinfected serum in 250 µl aliquots was then added to a Protein G IgG purification spin column (Thermo Scientific, UK), washed to remove all detergent and IgG eluted, as per manufacturer’s protocol, in 1.2 ml of neutralized elution buffer. The final concentration of IgG was determined using a Nanodrop 1000 spectrophotometer (Thermo Scientific, UK).

***Cell lines***

Human hepatoma Huh-7 cells and human epithelial kidney (HEK)-293T cells were grown in Dulbecco’s modified Eagle’s medium supplemented with 10% fetal calf serum, 5% non-essential amino acids, and 200 mM L-glutamine (Invitrogen).

***Generation of HCV pseudoparticles***

HEK-293T cells in subconfluent 10 cm tissue culture dishes were co-transfected with the retrovirus packaging vector pMLV gag – pol, the transfer vector pMLV-Luc and the HCV E1E2-expressing vector phCMVcE1E2. After 24 hours medium was replenished with 6 ml of DMEM containing 10% FCS. After 72 hours, the supernatant medium was harvested and passed through a 0.45 µM filter and used as a source of HCVpp. Pseudoparticles incorporating E1E2 from HCV genotype (gt) 1a (strain H77c, accession number AF011751.1), and 3a (sequence UKN3a1.28 F4/2-35; closely related to accession number AY734984.1) were generated. Particles devoid of HCV glycoproteins were also generated as negative control.

***E1E2 GNA Capture ELISA***

Briefly, Immulon II ELISA plates (Dynal, Stone, United Kingdom) coated with *Galanthus nivalis* agglutinin (GNA) were used to capture E1E2 glycoproteins from lysates of HEK-293T cells transfected with plasmids expressing E1E2 glycoproteins. IgG for each EU, CHCV and HC subject was subsequently added at a concentration of 200 µg/ml in PBS containing 0.05% Tween-20 and 2% Skimmed Milk Powder (PBSTM). After incubation and wash steps, the bound human IgGs were detected using an HRP conjugated anti-human IgG antibody (Sigma A0170) diluted 1:5000 and TMB (3,3’, 5, 5’-tetramethylbenzidine, Sigma) substrate. Absorbance values were measured at 450 nm using a Varioskan plate reader.

***Purified E2 IgG and IgM ELISA***

A purified soluble form of gt 1a E2 (H77) protein (sE2) was obtained following expression in High Five insect cells (Life Technologies Ltd., Paisley, UK). Immulon II ELISA plates were coated with 100 µl per well of sE2 at concentration of 1 µg/mL. Plates were washed and 50 µl of serum diluted 1:50 in PBSTM was added to each well in duplicate. After washing, binding of human IgG was detected as described for the GNA ELISAs.

To detect IgM, Immulon II ELISA plates were coated with sE2 as described above. Subject serum was diluted 1:50 with PBSTM and pre-incubated three times on a glutathione-S-transferase coated plate to remove non-specific antibodies, then added to the E2-coated plate. After incubation and wash steps using high tween PBSTM (0.5% Tween), IgM binding was detected using HRP-conjugated anti-Mu antibody (Abcam). The remainder of the ELISA protocol was as for the E1E2 ELISA described above.

**
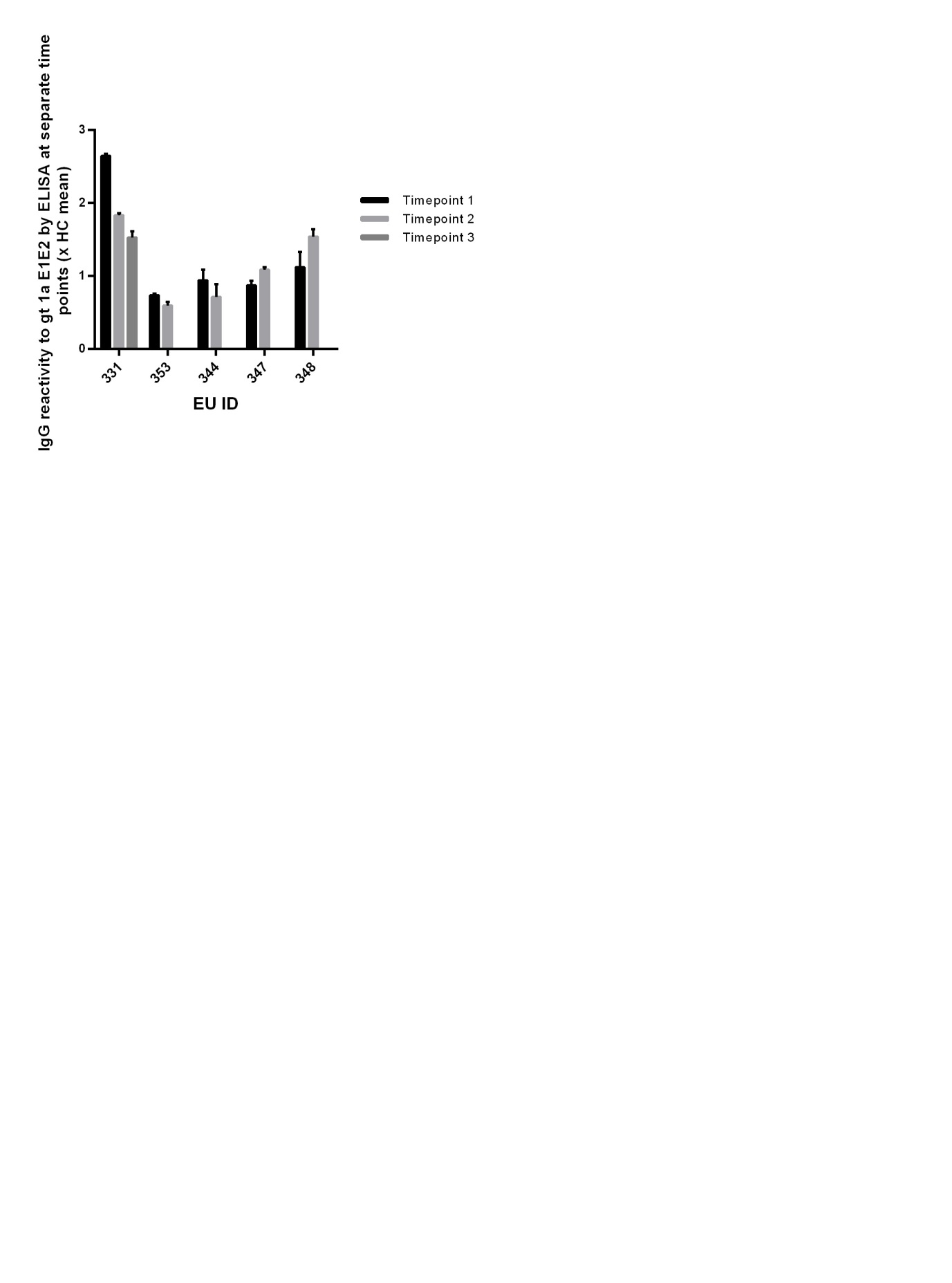
**

**Supplementary Fig. 1.** **Anti-E1E2 responses over time.** Binding of EU IgG to gt 1a E1E2 for the 5 individuals with samples from multiple time points ≥1 year apart. Error bars represent SEM. There were no significant differences between values at different time points for each individual, although a trend towards lower values is seen in later time points for 331.

**Supplementary** **Table 1: Binding sites of conformational antibodies used in competition ELISA**

| Antibody | Binding site (aa) | Reference |
| --- | --- | --- |
| **CBH7 (neutralizing)** | **540** | **[1]** |
| **HC1 (neutralizing)** | **523-535** | **[2]** |
| **HC11 (neutralizing)** | **412-423, 523-535** | **[3]** |
| **CBH4B (non-neutralizing)** | **549, 587** | **[4]** |

**Supplementary Table 2: IgG ELISA and Neutralization assay results for individual EU subjects.**

| **EU ID** | **x HC g1a E1E2 ELISA** | **x HC g3a E1E2 ELISA** | **x HC sE2 ELISA** | **Neutralisation >50%**  **Gt 1 Gt 3** | | **Neutralisation >40%**  **Gt 1 Gt 3** | |
| --- | --- | --- | --- | --- | --- | --- | --- |
| 246 | 1.2 | 1.3 | 1.9 |  |  |  |  |
| **306** | 2.2 | 1.5 | 2.3 | Y | Y | Y | Y |
| **307** | 1.8 | 1.2 | 2.4 | Y | Y | Y | Y |
| **315** | 1.3 | 1.1 | 2.5 | Y |  | Y |  |
| **318** | 1.4 | 2.5 | 1.3 |  |  |  |  |
| 320 | 1.4 | 1.2 | 0.9 |  |  |  |  |
| 324 | 1.8 | 1.4 | 1.2 |  |  |  |  |
| **331** | 2.6 | 1.7 | 3.9 | Y |  | Y | Y |
| 333 | 1.3 | 1.2 | 0.8 |  |  |  |  |
| **352** | 1.0 | 1.7 | 2.6 |  |  |  |  |
| 353 | 0.7 | 1.1 | 1.5 |  |  |  |  |
| **354** | 1.8 | 1.2 | 3.5 |  |  |  |  |
| **447** | 1.9 | 1.0 | 2.2 |  |  |  |  |
| **461** | 2.6 | 1.5 | 1.7 |  |  | Y | Y |
| 466 | 1.0 | 0.8 | 1.1 |  |  |  |  |
| 270 | 1.3 | 1.6 | 1.0 |  |  |  |  |
| 298 | 1.2 | 1.4 | 0.8 |  |  |  |  |
| **301** | 1.8 | 1.5 | 2.9 |  |  | Y | Y |
| 303 | 1.3 | 0.9 | 1.3 |  |  |  |  |
| 304 | 0.7 | 1.1 | 1.5 |  |  |  |  |
| 309 | 1.7 | 0.9 | 1.3 |  |  |  |  |
| 319 | 0.9 | 0.9 | 1.8 |  |  |  |  |
| **222** | 2.2 | 1.1 | 0.7 |  |  |  |  |
| 326 | 1.2 | 1.2 | 1.7 |  |  |  |  |
| 332 | 0.9 | 1.0 | 1.8 |  |  |  |  |
| 344 | 0.9 | 1.1 | 0.8 |  |  |  |  |
| **345** | 1.1 | 1.2 | 9.1 |  |  | Y |  |
| 347 | 1.1 | 0.8 | 2.0 |  |  |  |  |
| 350 | 0.5 | 1.7 | 0.9 |  |  |  |  |
| 444 | 1.0 | 1.3 | 1.9 |  |  |  |  |
| **445** | 1.6 | 2.4 | 0.8 |  |  |  |  |
| **446** | 2.5 | 1.2 | 2.4 |  |  |  |  |
| **458** | 2.1 | 3.9 | 1.7 | Y |  | Y | Y |
| **459** | 0.6 | 0.8 | 2.0 |  |  |  |  |
| **469** | 0.8 | 0.6 | 2.9 |  |  |  |  |
| **257** | 4.0 | 1.7 | 3.2 | Y | Y | Y | Y |
| 290 | 1.9 | 1.4 | 0.7 |  |  |  |  |
| 294 | 1.2 | 1.2 | 1.0 |  |  |  |  |
| 346 | 1.6 | 1.3 | 1.3 |  |  |  |  |
| **348** | 1.5 | 1.2 | 2.2 |  |  |  |  |
| **349** | 1.6 | 1.6 | 2.1 |  |  |  |  |
| 456 | 0.9 | 0.8 | 0.9 |  |  |  |  |

**Key:**

Values for ELISA are absorbances as multiples of the HC mean for each assay. In neutralization columns, Y indicates HCVpp infectivity was reduced by >50 or >40% respectively in the presence of 400µg/ml of purified EU IgG. Values in dark grey reach the specified cut off. Values in light grey are higher than HC mean plus 2 standard deviations but do not reach the 2× HC cut off. As only a selection of samples were tested in the neutralization assays, a black box indicates the sample was not tested.

**References**

1 Keck ZY, Li TK, Xia J*, et al.* Analysis of a highly flexible conformational immunogenic domain a in hepatitis C virus E2. *J Virol* 2005; 79(21):13199-13208.

2 Keck ZY, Saha A, Xia J*, et al.* Mapping a region of hepatitis C virus E2 that is responsible for escape from neutralizing antibodies and a core CD81-binding region that does not tolerate neutralization escape mutations. *J Virol* 85(20):10451-10463.

3 Eren R, Landstein D, Terkieltaub D*, et al.* Preclinical evaluation of two neutralizing human monoclonal antibodies against hepatitis C virus (HCV): a potential treatment to prevent HCV reinfection in liver transplant patients. *J Virol* 2006; 80(6):2654-2664.

4 Keck ZY, Xia J, Cai Z*, et al.* Immunogenic and functional organization of hepatitis C virus (HCV) glycoprotein E2 on infectious HCV virions. *J Virol* 2007; 81(2):1043-1047.
